# Supplementary figures and images for: Targeting of chondrocyte plasticity via connexin43 modulation attenuates cellular senescence and fosters a pro-regenerative environment in osteoarthritis
Source: Cell Death Dis. 2018 Dec 5;9(12):1166. doi: 10.1038/s41419-018-1225-2 (PMC6281585; doi:10.1038/s41419-018-1225-2)

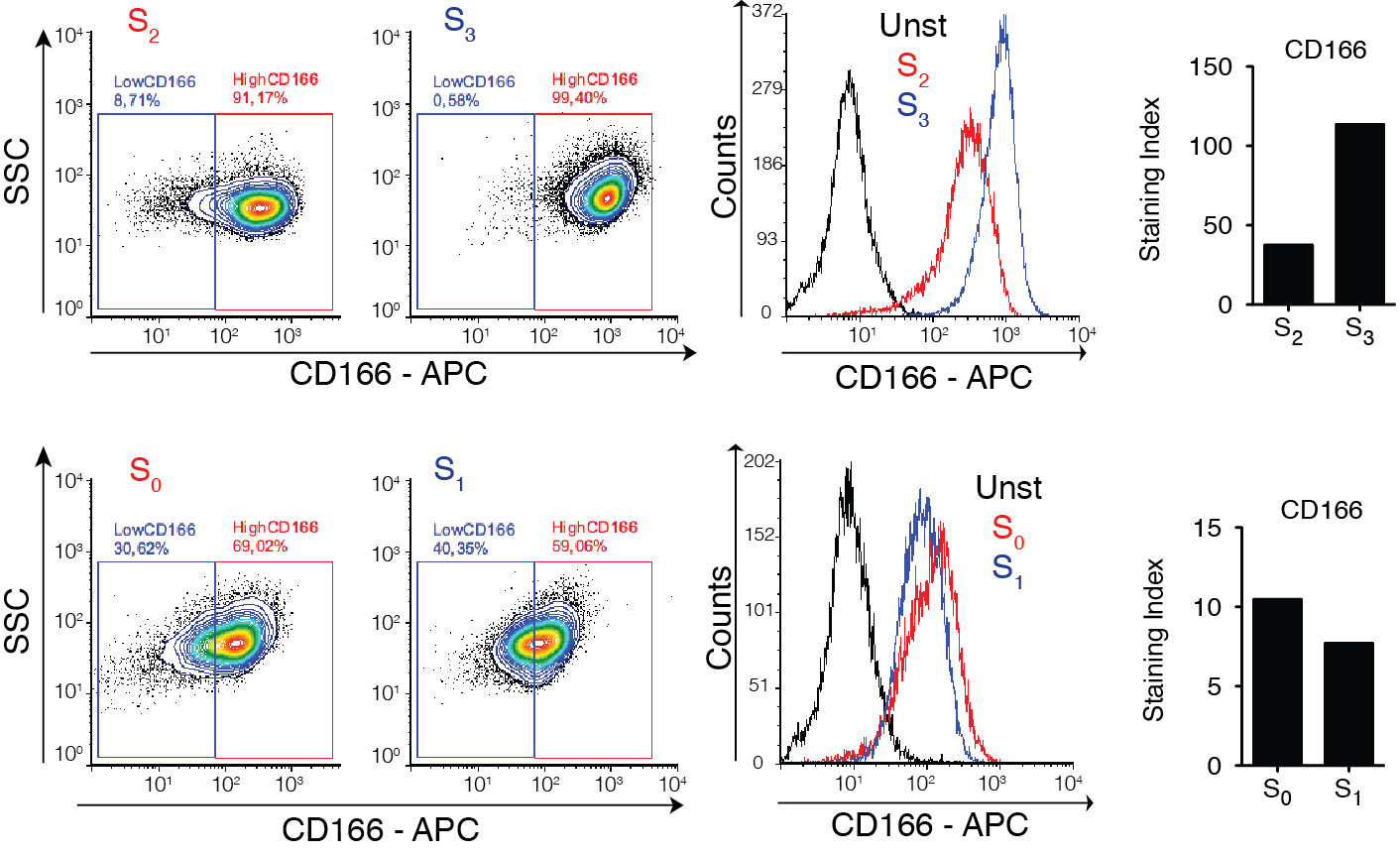

Supplement: Supplementary file 1 — Figure S1 [file 41419_2018_1225_MOESM1_ESM.tif]

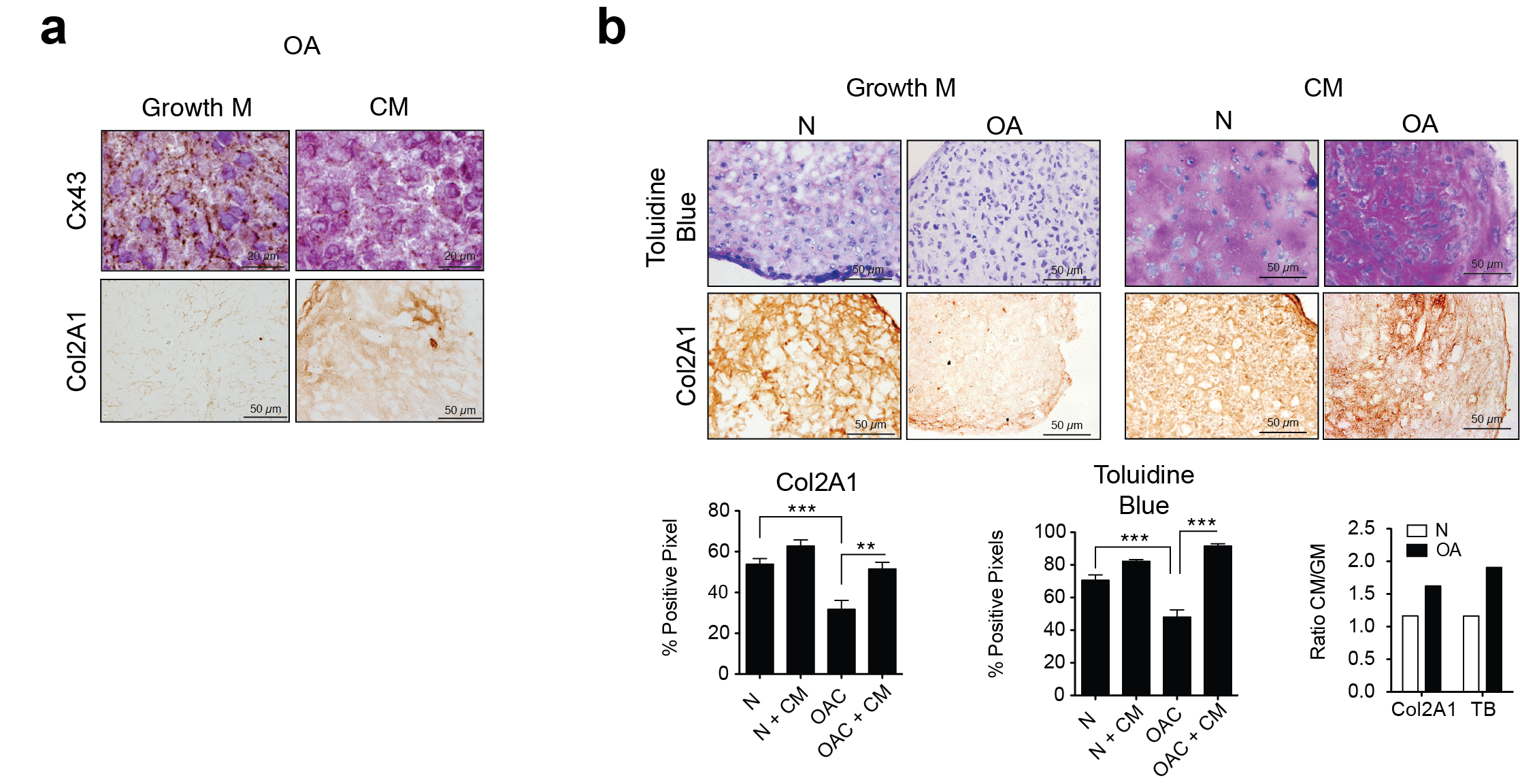

Supplement: Supplementary file 2 — Figure S2 [file 41419_2018_1225_MOESM2_ESM.tif]

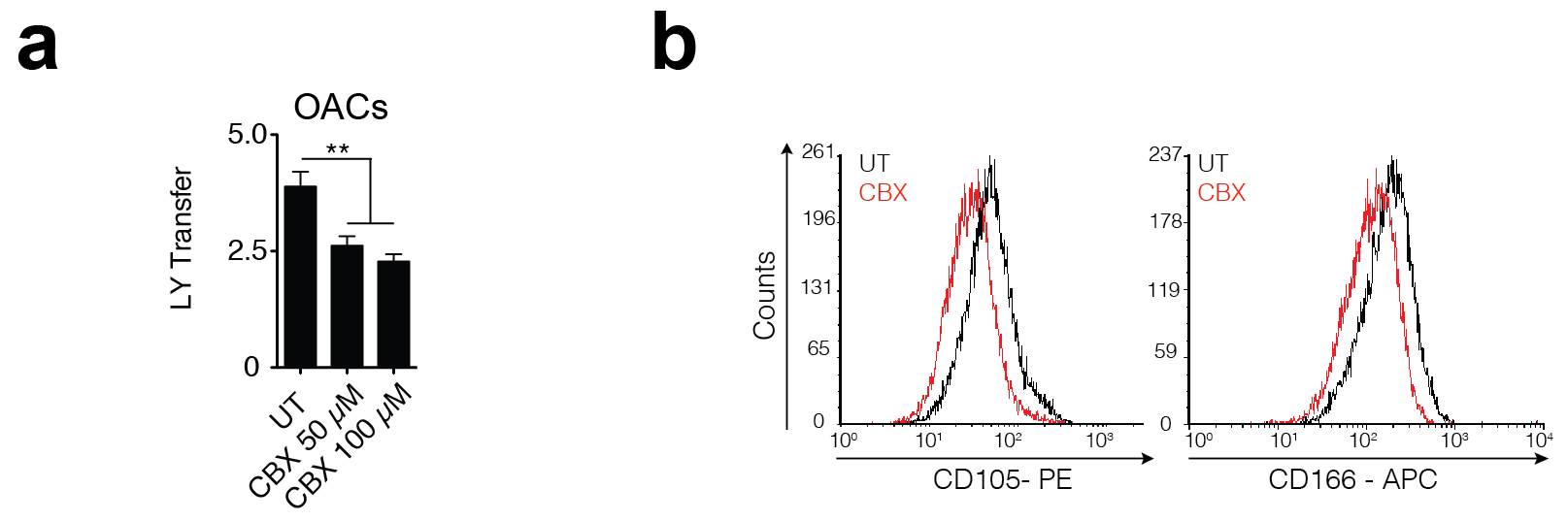

Supplement: Supplementary file 3 — Figure S3 [file 41419_2018_1225_MOESM3_ESM.tif]

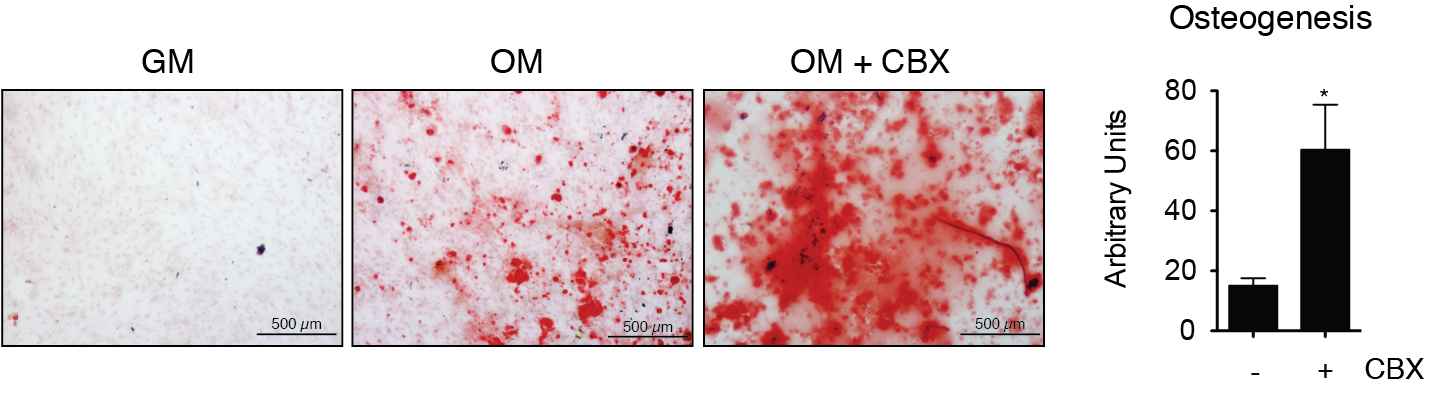

Supplement: Supplementary file 4 — Figure S4 [file 41419_2018_1225_MOESM4_ESM.tif]

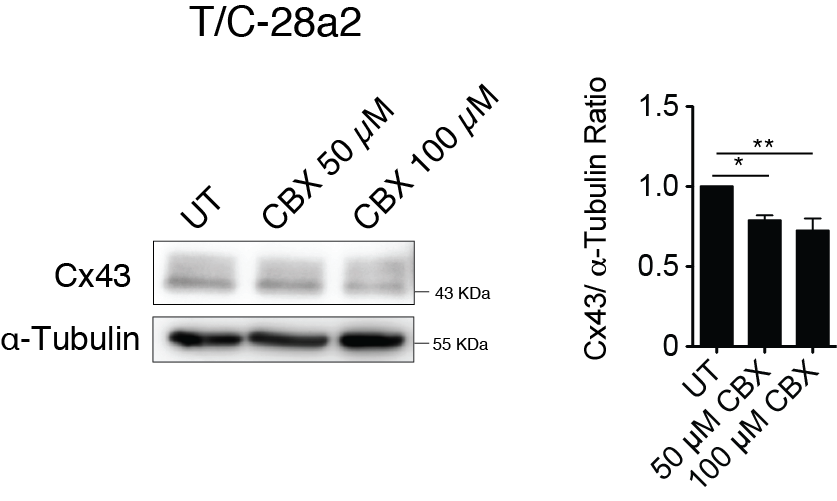

Supplement: Supplementary file 5 — Figure S5 [file 41419_2018_1225_MOESM5_ESM.tif]

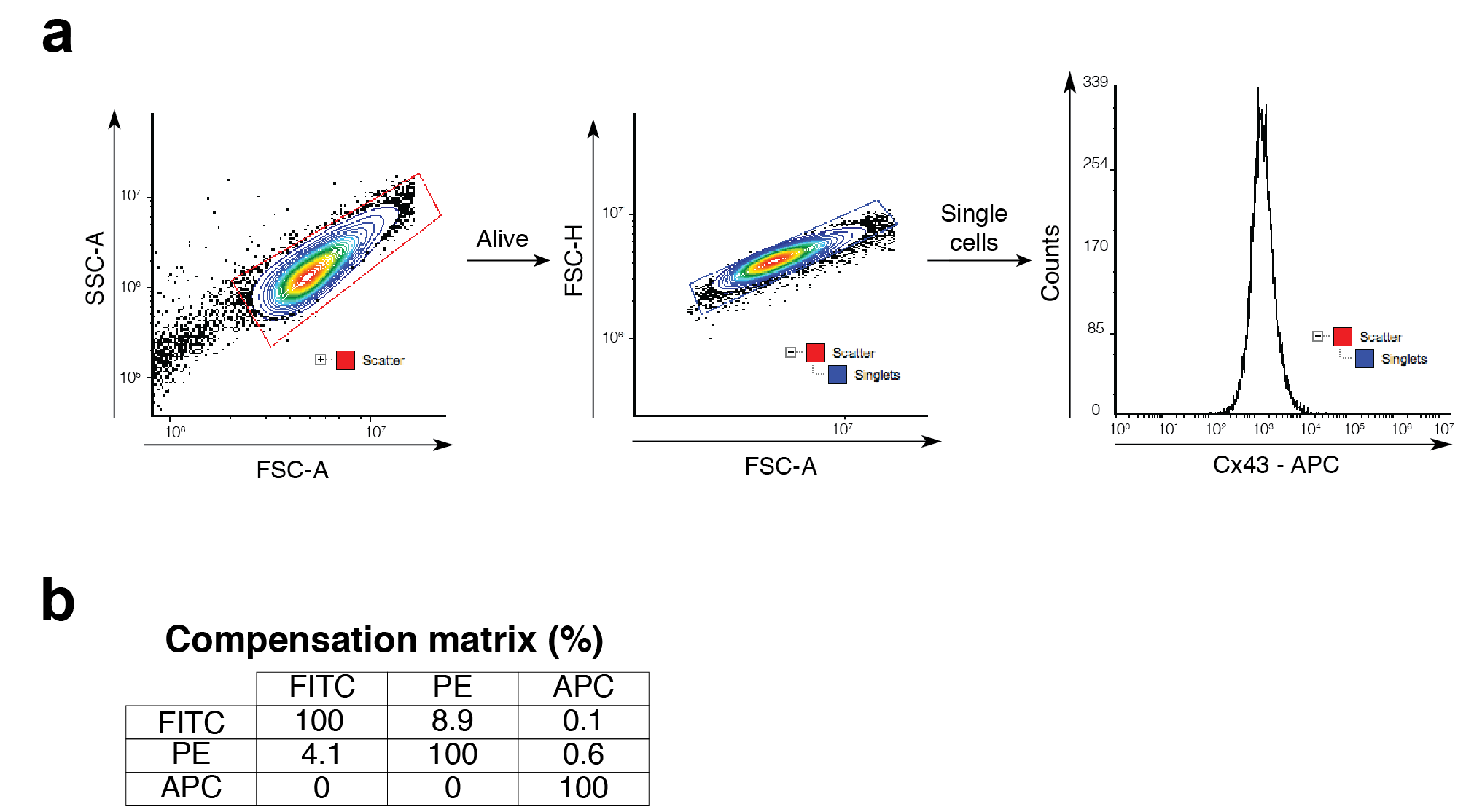

Supplement: Supplementary file 6 — Figure S6 [file 41419_2018_1225_MOESM6_ESM.tif]
