# Supplementary material for: Targeting of chondrocyte plasticity via connexin43 modulation attenuates cellular senescence and fosters a pro-regenerative environment in osteoarthritis
Source: Cell Death Dis. 2018 Dec 5;9(12):1166. doi: 10.1038/s41419-018-1225-2 (PMC6281585; doi:10.1038/s41419-018-1225-2)
Supplement: Supplementary file 7 — Supplementary Table 1 [file 41419_2018_1225_MOESM7_ESM.pdf]

Suplementary table 1.

Gene name (protein name)	Forward	Reverse
Cdkn2a (p16^{Ink4a})	GAGCAGAACGATAGGGCTTG	CATGTGCCCTCTCCTCCTAA
ALCAM (CD166)	TTCCAGAACACGATGAGGCA	GGACAACTAGGACAGTTTCTCT
GJA1 (Cx43)	ACATGGGTGACTGGAGCGCC	ATGATCTGCAGGACCCAGAA
HPRT-1 (HPRT-1)	TTGAGTTTGGAAACATCTGGAG	GCCCAAAGGGAACTGATAGTC
IL-1ß (IL-1ß)	CGAATCTCCGACCACCACTAC	TCCATGGCCACAACAACTGA
IL-6 (IL-6)	TGTAGCCGCCCCACACA	GGATGTACCGAATTTGTTTGTA
MMP-3 (MMP-3)	CCCTGGGTCTCTTTCACTCA	GCTGACAGCATCAAAGGACA
MMP-13 (MMP-13)	GGTTCCTGATGTGGGTGAAT	AGAAGTCGCCATGCTCCTTA
CDH2 (N-cadherin)	TATTTCCATCCTGCGTGTGA	GCGTTTCATCCATACCACAA
PTGS2 (COX-2)	CTTCACGCATCAGTTTTTCAAG	TCACCGTAAATATGATTTAAGTCCAC
TWIST1 (Twist-1)	CATGTCCGCGTCCCACTA	CACGCCCTGTTTCTTTGAAT
VIM (Vimentin)	ACTTTGCCGTTGAAGCTG	AATCCAGATTAGTTTCCCTCAGGT
