# Supplementary material for: Targeting of chondrocyte plasticity via connexin43 modulation attenuates cellular senescence and fosters a pro-regenerative environment in osteoarthritis
Source: Cell Death Dis. 2018 Dec 5;9(12):1166. doi: 10.1038/s41419-018-1225-2 (PMC6281585; doi:10.1038/s41419-018-1225-2)
Supplement: Supplementary file 8 — Supplementary figure legends [file 41419_2018_1225_MOESM8_ESM.docx]

**Supplementary Figure Legends**

**Supplementary Figure 1.** Images showing CD166 levels and population detected by flow cytometry from two different OAC samples after monolayer culture. Dedifferentiation detected by increased levels of CD166 is correlated with the number of passages in culture (S_0_–S_3_).

**Supplementary Figure 2.** (**a**) Immunohistochemistry of Cx43 and Col2A1 in OACs cultured as a micromass pellet for 30 days in the presence of normal growth medium (Growth M) or in chondrogenic medium (CM). (**b**) Comparative images of OACs and healthy chondrocytes (N) in a 3D culture in chondrogenic medium (CM) versus growth medium. Chondrogenesis was evaluated by the levels of PGs (toluidine blue staining) and Col2A1. Quantification for Col2A1 and Toluidine Blue are shown below (n=5; mean±s.e.m.; ***P*<0.01, ****P*<0.0001; one-way ANOVA). The ratio (fold change) of Col2A1 and Toluidine Blue in OACs and healthy chondrocytes in growth medium and CM are showed on the right.

**Supplementary Figure 3.** (**a**) The treatment of OACs with 50 and 100 µM CBX for 15 min significantly reduced the levels of GJIC (n=5; mean±s.e.m.; ***P*<0.01; Mann–Whitney test). (**b**) Flow cytometry analysis of CD105 and CD166 antigens detected in OACs treated with 100 µM CBX for 7 days. Quantification is shown in Fig. 2c.

**Supplementary Figure 4.** (**a**) OACs were cultured in osteogenic medium (OM) and supplemented with 50 µM CBX for 21 days. The osteogenesis was evaluated by the alizarin red staining positivity (n=6, mean±s.e.m.; **P*<0.05; Mann–Whitney test).

**Supplementary Figure 5.** Detection of Cx43 levels by western blot in T/C-28a2 cells cultured in normal medium (UT, DMEM 10% FBS) and treated with 50 and 100 µM CBX for 48 hours (n=4; mean±s.e.m.; **P*<0.05, ***P*<0.01; one-way ANOVA).

**Supplementary Figure 6.** (**a**) Gating strategy used in flow cytometry analysis. A single living population was selected by the forward scatter (FSC) and side scatter (SSC) properties of the cells. Cell aggregate discrimination was performed by comparing area versus high signal in FSC. (**b**) The compensation matrix used for FITC-PE-APC analysis in a BD Accuri C6 (Becton Dickinson) flow cytometer.

**Supplementary Table 1**. List of primer sequences (5'–3') used for RT-PCR analysis.
